# Highly heterogeneous residual malaria risk in western Thailand

**DOI:** 10.1016/j.ijpara.2019.01.004

**Published:** 2019-05

**Authors:** Wang Nguitragool, Stephan Karl, Michael White, Cristian Koepfli, Ingrid Felger, Pratap Singhasivanon, Ivo Mueller, Jetsumon Sattabongkot

**Affiliations:** a Department of Molecular Tropical Medicine & Genetics, Faculty of Tropical Medicine, Mahidol University, Bangkok, Thailand; b Population Health and Immunity Division, Walter and Eliza Hall Institute of Medical Research, Parkville, Victoria, Australia; c Department of Medical Biology, University of Melbourne, Parkville, Victoria, Australia; d Vector-borne Diseases Unit, Papua New Guinea Institute of Medical Research, Madang, Madang Province, Papua New Guinea; e Malaria: Parasites and Hosts Unit, Department of Parasites & Insect Vectors, Institute Pasteur, Paris, France; f Department of Medical Parasitology and Infection Biology, Swiss Tropical & Public Health Institute, Basel, Switzerland; g Department of Tropical Hygiene, Faculty of Tropical Medicine, Mahidol University, Bangkok, Thailand; h Mahidol Vivax Research Unit, Faculty of Tropical Medicine, Mahidol University, Bangkok, Thailand

**Keywords:** Malaria, Epidemiology, *Plasmodium vivax*, *Plasmodium falciparum*, Force of infection, Prevalence, Incidence, Thailand

## Abstract

Over the past decades, the malaria burden in Thailand has substantially declined. Most infections now originate from the national border regions. In these areas, the prevalence of asymptomatic infections is still substantial and poses a challenge for the national malaria elimination program. To determine epidemiological parameters as well as risk factors for malaria infection in western Thailand, we carried out a cohort study in Kanchanaburi and Ratchaburi provinces on the Thailand-Myanmar border. Blood samples from 999 local participants were examined for malaria infection every 4 weeks between May 2013 and Jun 2014. Prevalence of *Plasmodium falciparum* and *Plasmodium vivax* was determined by quantitative PCR (qPCR) and showed a seasonal variation with values fluctuating from 1.7% to 4.2% for *P. vivax* and 0% to 1.3% for *P. falciparum.* Ninety percent of infections were asymptomatic. The annual molecular force of blood-stage infection (_mol_FOB) was estimated by microsatellite genotyping to be 0.24 new infections per person-year for *P. vivax* and 0.02 new infections per person-year for *P. falciparum*. The distribution of infections was heterogenous, that is, the vast majority of infections (>80%) were found in a small number of individuals (<8% of the study population) who tested positive at multiple timepoints. Significant risk factors were detected for *P. vivax* infections, including previous clinical malaria, occupation in agriculture and travel to Myanmar. In contrast, indoor residual spraying was associated with a protection from infection. These findings provide a recent landscape of malaria epidemiology and emphasize the importance of novel strategies to target asymptomatic and imported infections.

## Highlights

• There is a highly heterogenous risk of malaria infection among villagers in western Thailand.
• The molecular force of infection was determined in a low endemic setting.
• There is a strong correlation between malaria prevalence and the force of infection.

## Graphical abstract

**Figure:**
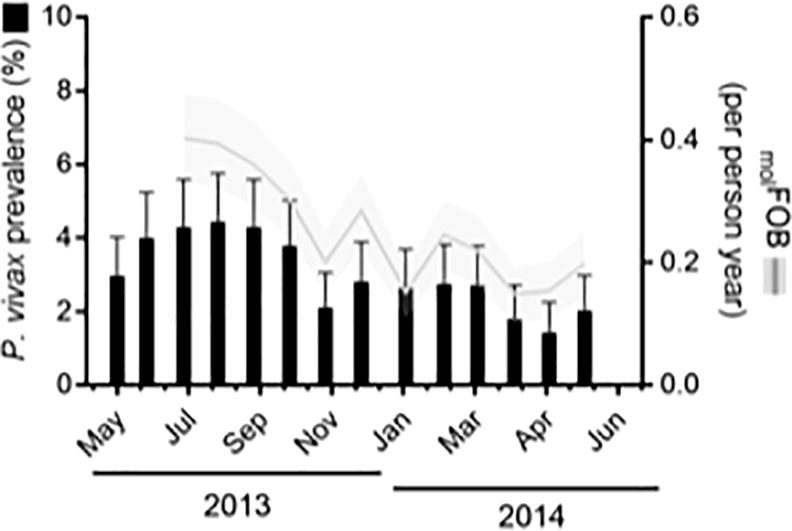

## 1 Introduction

In recent years, significant progress has been made in controlling malaria worldwide. The malaria incidence and mortality rates of Southeast Asia declined by approximately 50% between 2000 and 2015 (WHO, 2015). In the same period, the number of malaria cases in Thailand was reduced from 150,000 to 24,850 (ThaiDDC, 2016). The incidence rate in most areas is now below 1 case per 1000 person-years at risk. As part of the global malaria elimination effort, the country is aiming to achieve malaria elimination before 2025 (WPRO, 2015), a commendable goal which will require commitment from all parties involved.

Notwithstanding the recent success in reducing the malaria burden, a significant proportion of the population living in endemic areas of Thailand is still at risk of *Plasmodium* spp. infections. Historically, *Plasmodium falciparum* was the predominant parasite species in Thailand, but *Plasmodium vivax* has recently taken over as the primary parasite whereas *Plasmodium malariae*, *Plasmodium ovale* and *Plasmodium knowlesi* are only found sporadically (ThaiMoPH, 2017). Malaria infections in Thailand are seasonal, the peak season lasting from May to September (Phimpraphi et al., 2008). Transmission is concentrated along the western Myanmar border and the eastern Cambodian border. Cross border movements are thought to contribute significantly to maintenance of the disease (Sriwichai et al., 2017). *Anopheles dirus*, *Anopheles minimus* and *Anopheles maculatus* are the most important local malaria vectors in these parts of the country (Tananchai et al., 2012, Sriwichai et al., 2016). Recently, an increasing number of clinical infections were also reported from the southern provinces (ThaiMoPH, 2017) which is likely due to reduced surveillance and control activities as a result of political unrest in the area.

Several studies have reported the presence of a large number of asymptomatic infections in Thailand (Li et al., 2014, Baum et al., 2015, Baum et al., 2016, Parker et al., 2015, Nguitragool et al., 2017). As most asymptomatic carriers do not seek treatment, they are a neglected reservoir that may help sustain disease transmission (Kiattibutr et al., 2017). To accelerate progress towards elimination, it is important for the national malaria program to have an appropriate strategy to manage these infections. A better understanding of which individuals are at the greatest risk of harboring infections, symptomatic or asymptomatic, is thus of fundamental importance, especially when resources for malaria control are limited. At present, the Thai national malaria control program relies mostly on clinical cases detected at health facilities as the main indicator of malaria incidence to coordinate its control efforts. Data on asymptomatic infection rates in Thailand are thus restricted to cross-sectional surveys conducted in a few areas (Kritsiriwuthinan and Ngrenngarmlert, 2011, Li et al., 2014, Baum et al., 2015, Baum et al., 2016, Parker et al., 2015).

In this study, we sought to better understand the dynamics of malaria infection and its risk factors in populations along the Thailand-Myanmar border, an area where malaria infection rates are still substantially higher than in other parts of Thailand. We conducted a longitudinal cohort study in two villages between May 2013 and Jun 2014 in which finger-prick blood samples were obtained from participants every 4 weeks. Malaria parasites were detected by qPCR and parasite genotypes were assessed by amplification of length polymorphic markers. Parasite positivity and molecular force of blood-stage infection (_mol_FOB, the rate of acquisition of new blood-stage clones) (Mueller et al., 2012, Koepfli et al., 2013) were used to quantify the risk of infection as well as the underlying risk factors.

## 2 Materials and methods

### 2.1 Study sites

**Fig. 1:**
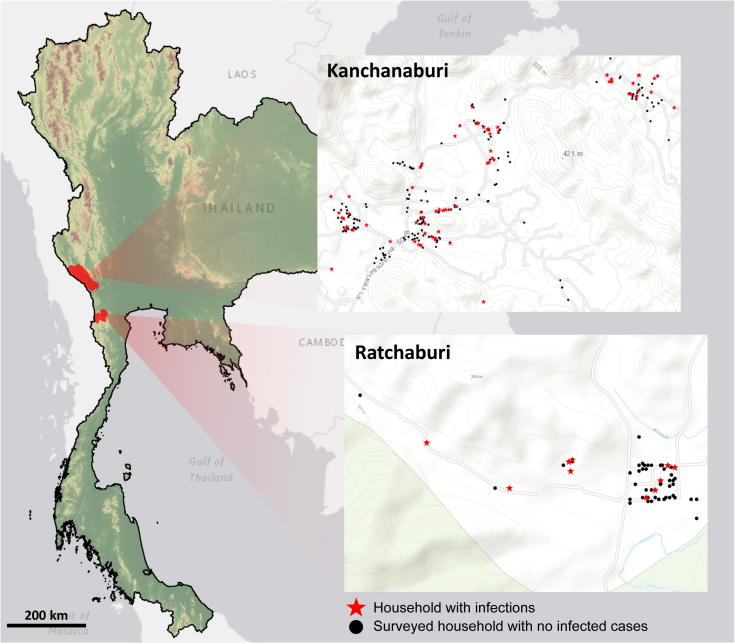
Map of the two study sites in Bong Ti, Kanchanaburi and Suan Phueng, Ratchaburi on the Thailand-Myanmar border. Study households are shown (●) as well as households in which infections with *Plasmodium falciparum* or *Plasmodium vivax* were detected over the course of the study (★).

This study was conducted in two villages, one in the Bong Ti subdistrict of Kanchanaburi province and the other in the Suan Phueng subdistrict of Ratchaburi Province of Thailand (Fig. 1). These two sites are located within a few km of the Thailand-Myanmar border. The main ethnic groups in both sites were Thai and Karen. Agriculture and farming were the main occupations. Forest foraging was a frequent activity for residents in these areas. The village of Bong Ti is located approximately 65 km west of the main district of Kanchanaburi province in a hilly terrain. Most houses were accessible by paved roads. A cross-sectional survey of malaria infection by qPCR (Nguitragool et al., 2017) indicated 3.8% *Plasmodium vivax* and 1.4% *P. falciparum* prevalence in August-September of 2012. The study village in Suan Phueng subdistrict is located approximately 160 km from Bangkok. The village is located in a mountainous area on the Tanaosri mountain range. Our survey in September-October of 2012 indicated a prevalence of 3.1% for *P. vivax* and 1.2% for *P. falciparum* at this site (Nguitragool et al., 2017).

### 2.2 Study design

A total of 999 participants were enrolled for a 1 year prospective cohort study. No treatment was given at the baseline. A total of 14 visits to each study participant were made between May 2013 and June 2014, with time between consecutive visits being approximately 4 weeks. A finger prick blood sample was collected from each participant at each visit for malaria parasite detection, species identification, and genotyping by length polymorphic markers for _mol_FOB determination. Additional finger prick blood samples were also obtained through passive case detection, when participants visited malaria clinics in the study areas during the time between study visits.

At each visit, participants were asked to provide information regarding factors relevant for malaria control including access to and usage of insecticide-treated nets, travel history, clinical malaria history, and treatment taken since the last visit. Before taking the blood sample, the body temperature of each participant was measured using an infrared thermometer. Participants with fever (body temperature ≥37.5 °C) were tested for malaria infection on-site using a rapid diagnostic test (SD BIOLINE Malaria Ag P.f./Pan, Standard Diagnostics, Republic of Korea) and referred to a nearby malaria clinic for treatment if the test was positive. The study was approved by the Ethics Committee of the Faculty of Tropical Medicine, Mahidol University, Thailand (EC approval number TMEC 13-020).

### 2.3 Molecular analysis

Finger prick whole blood (200 μl) was collected from each participant into an EDTA-containing microtainer. After plasma removal, blood pellets were stored at −20 °C until DNA extraction. DNA was extracted using FavorPrep 96-well Genomic DNA Extraction Kits (Favorgen, Taiwan), following the standard protocol provided by the manufacturer. The final purified DNA was eluted into 100 μl of elution buffer. A volume of 4 μl of purified DNA (equivalent to 8 μl of whole blood) was used as the template for malaria parasite detection by a genus-specific qPCR assay targeting the *Plasmodium* 18S rRNA genes (Wampfler et al., 2013). For samples positive by the genus-specific assay, *P. vivax-* and *P. falciparum*-specific assays (Rosanas-Urgell et al., 2010) were used to determine the parasite species. *Plasmodium vivax* genotypes were determined using PvMS2 and Pvmsp1F3 markers as previously described (Schoepflin et al., 2009). The combined expected heterozygosity, *H*_e_, of the two markers was 0.89. Capillary electrophoresis for microsatellite genotyping was performed on an ABI3730XL instrument (Macrogen, Republic of Korea).

### 2.4 Determination of the molecular force of blood infection (_mol_FOB)

The molecular force of blood infection (_mol_FOB) was determined as described previously (Koepfli et al., 2013). Briefly, the _mol_FOB is the observed number of new blood-stage infections, as identified by individual parasite genotypes, divided by the time-at-risk (i.e., the incidence of new blood-stage infections). An observed infection, as identified by a specific genotype, was considered to be ‘new’ if the same genotype had not been seen in the two previous active or passive case detection visits. As such, the _mol_FOB can be determined for each study participant or as a sum over the entire study population or subpopulations.

### 2.5 Statistical analysis

Factors influencing i) the overall rate of acquisition of new blood infections (_mol_FOB); ii) being parasite-positive for *P. vivax* and/or *P. falciparum* and iii) being subject to a clinical episode, defined by fever + parasitemia detectable by light microscopy (Robinson et al., 2015) were investigated using statistical models. Travel to Myanmar was quantified as ‘trips per year’ based on answers to questions about travel history at each visit. Since the _mol_FOB is a count variable measured per individual over a specific exposure time (time at risk), and is overdispersed, a negative binomial regression model was chosen in which the exposure time at risk is used as tge offset (µ*_j_ = exp(βx_j_ + offset_j_ + ν_j_), where offset_j_ = log(exposure time) and e^νj^ follows a gamma distribution. Because using the collapsed data to model the _mol_FOB for each individual does not allow for the analysis of time-changing covariates, factors influencing the frequency of parasite positivity and frequency of clinical episodes within the study period were explored using multiple failure time models allowing for time-changing covariates (Therneau and Grambsch, 2000). In these models, parasite positivity and clinical episodes were equivalent to a ‘failed’ outcome, respectively. In addition to the statistical models presented in the main manuscript, univariate analyses (Supplementary Tables S1, S3, S5, S7) and multivariate analyses with backward selection (Supplementary Tables S2, S4, S6) are provided.

To test whether infections were spatially correlated, the _mol_FOB was tested for spatial autocorrelation using the Moran’s I test statistic, which takes into account the value of the _mol_FOB for each location and the distance between all locations. A Morans I > 0 indicates spatial clustering of high/high and/or low/low _mol_FOB values. This analysis was done once for each individual, accepting that the distance between individuals in the same household is 0, and once for each household by calculating the average _mol_FOB per household (i.e., the total number of new infections divided by the total time at risk per household).

## 3 Results

### 3.1 Characteristics of the study population

In total, 999 participants were enrolled into this study in May 2013. Eight hundred and twelve participants were from Bong Ti, Kanchanaburi and 187 participants were from Suan Phueng, Ratchaburi. Fig. 1 shows the maps of these two study sites where the household locations of the study participants were marked.

Of the 999 participants, the large majority (*n* = 799, 80%) were seen 13 or more times (interquartile range (IQR): 13–14; range: 1–14) during the study period resulting in 12,559 blood samples from active case detection (ACD). In addition, 281 blood samples were obtained through passive case detection (PCD) when participants presented at local malaria clinics due to illness. The median time that the participants remained in the study was 368 days (IQR: 361–379; range: 0–378). An overview of the characteristics of the study population is given in Table 1.

**Table 1:** Characteristics of the western Thailand study population.

Characteristic	*n* (%) or median (range)
Age^a^ (yr)	23 (1–83)
Male/Female	452 (46.2)/527 (53.8)
Kanchanaburi/Ratchaburi	812 (81.3)/187 (18.7)
Exposure (days)	368 (22–378)
Infection status at first visit	
*Plasmodium vivax*	34 (3.4)
*Plasmodium falciparum*	7 (0.8)
Mixed *P. vivax*/*P. falciparum*	3 (0.3)

Insecticide treated net possession/usage^a^	
Never	62 (6.3)
<6 months	11 (1.1)
6 months–1 year	12 (1.2)
1 year–2 years	34 (3.5)
>2 years	860 (87.8)
Indoor residual spraying^a^	719 (73%)
Window screens^a^	20 (0.2%)

a At first visit (May 2013).

### 3.2 Parasite prevalence and _mol_FOB

From samples collected at both ACD and PCD visits, the genus-specific PCR assay detected 735 infections in the study participants, of which 512 (70.0%) were successfully genotyped for the _mol_FOB (i.e. the incidence of genetically distinct blood-staged infections) analysis. *Plasmodium vivax* monoinfections constituted 84.6% (*n* = 433/512), *P. falciparum* monoinfections 9.2% (*n* = 47/512) and mixed species infections 6.3% (*n =* 32/512). As such, mixed species infections were over-represented by a factor of approximately 10 (Fisher’s exact test, *P* < 0.001) indicating a strong clustering of infection. A proportion of 8.5% (37/433) of *P. vivax* infections and 21.3% (10/47) of the *P. falciparum* infections were classified as clinical, i.e., detectable parasitemia was accompanied by a measured or reported fever in either PCD or ACD visits. The difference is statistically significant (analysis of proportions, chi-square test, *P* = 0.006) and suggests that *P. falciparum* was more likely to cause clinical symptoms. Notably, none of the 32 mixed infections was classified as a clinical episode.

**Fig. 2:**
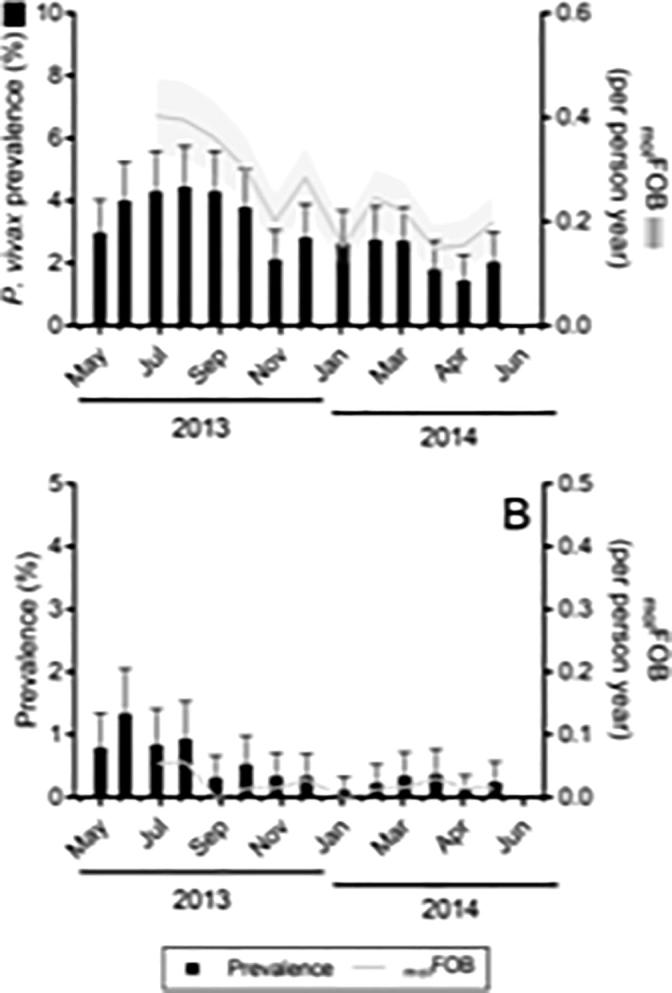
Prevalence and molecular force of blood-stage infection (_mol_FOB) of *Plasmodium vivax* (A) and *Plasmodium falciparum* (B) at each study site by month of study visit. Error bars (prevalence) represent the upper limit of the 95% confidence interval; shaded area (_mol_FOB) represents S.E.M. Note that _mol_FOB could not be determined for visits one and two as it depends on the observations from two previous study visits.

The prevalence of *P. falciparum* and *P. vivax* showed seasonal variation with prevalence varying from 4.2% to 1.7% for *P. vivax* and from 1.3% to 0% for *P. falciparum* (Fig. 2). The peak between June and September coincided with the rainy season.

During the study period, an estimated total of 226 new (i.e. genetically distinct) *P. vivax* and 18 new *P. falciparum* blood-stage infections were detected, resulting in an average _mol_FOB of 0.24 new infections per person-year for *P. vivax* and 0.02 new infections per person-year for *P. falciparum*. The seasonal variation of the _mol_FOB resembled that of parasite prevalence for both species, with the peak in the wet season.

### 3.3 Clustering of new infections

**Fig. 3:**
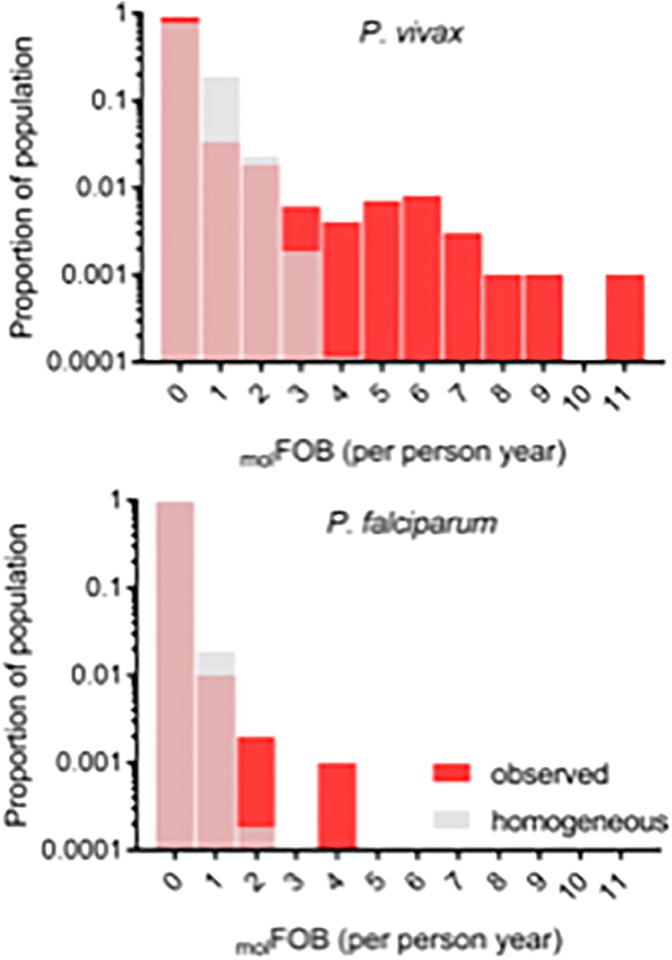
Distributions of the molecular force of blood-stage infection (_mol_FOB). The proportion of study participants at each level of _mol_FOB is shown for *Plasmodium vivax* (A) and *Plasmodium falciparum* (B). Red: normalized distributions of observed _mol_FOB in the study participants. Grey: Poisson distributions computed based on the mean _mol_FOB. The Poisson distributions are overlaid on top of the observed distributions.

A number of study participants experienced a disproportionately high _mol_FOB (Fig. 3). The excess of high _mol_FOB values compared with the homogeneous distribution (Poisson) is statistically significant for *P. vivax* (goodness of fit test for Poisson distribution *P* < 10^−6^), suggesting either a non-uniform risk of acquiring new infection through mosquito bites or relapses of parasites from the liver. Spatial analysis suggests that the *P. vivax*_mol_FOB in Bong Ti, Kanchanaburi was clustered at both the individual level (global Moran’s I = 0.12, *P* < 0.0001) and, more loosely, at the household level (global Moran’s I = 0.03, *P* = 0.02). The low number of infections precluded an in-depth analysis of heterogeneities in the risk of *P. falciparum* infection.

### 3.4 Factors associated with *P. vivax* infection and illness

**Fig. 4:**
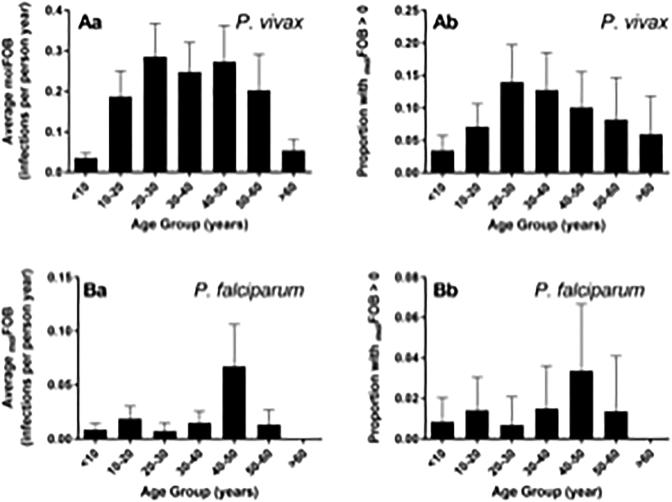
The molecular force of blood-stage infection (_mol_FOB) as a function of age in the study population. (Aa) The average *Plasmodium vivax*_mol_FOB in each age group. (Ab) The proportion of individuals with *P. vivax*_mol_FOB > 0 in each age group. (Ba) The average *Plasmodium falciparum*_mol_FOB in each age group. (Bb) The proportion of individuals with *P. falciparum*_mol_FOB > 0 in each age group. Error bars indicate the S.E.M. (Aa, Ba) or the upper limit of the 95% confidence interval (Ab, Bb).

The age profile of the _mol_FOB for *P. vivax* revealed a higher number of new blood-stage clones in adolescents and adults compared with children (Fig. 4). In addition to age, analysis of risk factors revealed that increased frequency of travel to Myanmar, previous malaria episodes, and employment in agriculture were significantly associated with increased risk of new *P. vivax* blood-stage infections as measured by the _mol_FOB (Table 2).

**Table 2:** Factors associated with *Plasmodium vivax*_mol_FOB (negative binomial model) in western Thailand.

Risk factor	aIRR	95% CI	*P*
Kanchanaburi	1.83	0.64–5.23	0.257

Age group (years; ref: 0–6)			
7–12	22.79	3.34–155.6	<0.001
13–17	17.10	2.15–135.84	
18–60	15.67	2.15–114.15	
>60	3.85	0.44–33.31	

Male	1.86	1.1–3.14	0.02
Frequency of travel to Myanmar (per visit/year)	1.15	1.05–1.24	0.001
Previous clinical malaria^a^	2.43	1.2–4.91	0.014
House treated with IRS^a^	0.56	0.3–1.04	0.067
Reported bednet possession^a^	0.98	0.39–2.43	0.957
Work in agriculture^a^	2.25	1.04–4.86	0.039

aIRR, adjusted incidence risk ratio; CI, confidence interval; IRS, indoor residual spray

a Status at enrolment.

In order to assess time changing covariates, risk factors associated with *P. vivax* positivity were also determined using a multiple failure time model (Table 3). In general, predictors of *P. vivax*_mol_FOB were predictors of *P. vivax* positivity. While similar effect sizes were observed, the analysis of *P. vivax* positivity additionally detected indoor residual spraying (IRS) as a significant factor associated with reduced risk of infection.

**Table 3:** Risk factors associated with western Thailand *Plasmodium vivax* positivity (multiple failure time model).

Risk factor	aHR	*P*
Kanchanaburi	2.22 (1.06–4.76)	0.03

Season (May-September)	3.41 (2.19–5.31)	<0.001

Age group (years; ref: 0–6)		
7–12	3.64 (0.94–14.02)	0.02
13–17	4.99 (1.22–20.34)	
18–60	3.69 (1.03–13.21)	
>60	0.99 (0.22–4.48)	

Male	2.32 (1.36–3.96)	<0.001
Work in agriculture^a^	2.01 (1.14–3.55)	0.02
Frequency of travel to Myanmar^b^	1.06 (1.01–1.11)	0.01
Average bednet usage^b^, ^c^	1 (0.95–1.05)	0.92
House treated with IRS^a^	0.47 (0.28–0.79)	<0.001
Previous clinical malaria^a^	1.87 (1.13–3.1)	0.02

aHR, adjusted hazard ratio; IRS, indoor residual spray.

a Status at enrolment.

b As a time-changing covariate (average observed at time of outcome).

c Average bednet usage was defined as the proportion of times a person had answered ‘yes’ to the question: ‘Did you sleep under a bednet last night?’ during active case detection.

Several predictors of *P. vivax* infection including season, age, occupation, and reported previous clinical malaria, were also significantly associated with clinical episodes of malaria (Table 4). Notably, while adult age was a predictor of increased risk of infection (Table 2, Table 3), it was associated with a reduced risk of clinical episodes (Table 4). This indicates stronger protective immunity in adults compared with young children. Although travel to Myanmar was a predictor of infection and IRS was associated with a reduced risk of infection, these two factors were not significantly associated with clinical episodes. This is consistent with their expected relationship with parasite exposure but not clinical outcomes. Interestingly, despite the lack of association of bednet usage with *P. vivax*_mol_FOB and positivity, bednet usage was significantly associated with a small reduction in clinical episodes. The reason for this is unclear and may deserve further investigation. None of the mixed species infections (*P. falciparum*/*P. vivax*, *n* = 32) corresponded to a clinical episode, possibly reflecting parasite-parasite interaction or co-development of host protective immunity against these sympatric species.

**Table 4:** Risk factors associated with western Thailand *Plasmodium vivax* clinical episodes (multiple failure time model).

Risk factor	aHR	*P*
Kanchanaburi	1.01 (0.3–3.33)	0.99

Season (May-September)	19.47 (1.82–208.79)	0.01

Age group (ref: 0–12)^b^		
13–17	0.75 (0.19–3.05)	<0.001
18–60	0.24 (0.1–0.6)	
>60	0.35 (0.09–1.29)	
Male	1.66 (0.63–4.36)	0.31
Work in agriculture^c^	4.64 (2.18–9.86)	<0.001
Travel frequency to Myanmar^a^	1.04 (0.94–1.15)	0.50
Average bednet usage^a^, ^d^	0.89 (0.83–0.96)	<0.001
IRS^c^	1.64 (0.61–4.45)	0.33
Previous clinical malaria^c^	2.21 (0.98–4.97)	0.06

aHR, adjusted hazard ratio; IRS, indoor residual spray.

a As a time-changing covariate (floating average at time of outcome).

b The age groups were further aggregated when compared with Table 3, as some age groups did not contain any positive outcomes.

c Status at enrolment.

d Average bednet usage was defined as the proportion of times a person had answered ‘yes’ to the question: ‘Did you sleep under a bednet last night?’.

### 3.5 Factors associated with *P. falciparum* infection

For *P. falciparum*, risk factors for increased _mol_FOB were not determined due to the small number of observations (*n* = 18 new *P. falciparum* infections out of 12,559 samples). Predictors of infection were determined only from positivity data. In the univariate analysis, only travel frequency to Myanmar was found to be a predictor of parasite positivity (Supplementary Table S7). The small number of detected *P. falciparum* infections did not allow detection of other risk factors. Based on the results presented in Table 5, however, season, age, male gender and IRS may also show association with a higher infection rate in a larger, more highly powered, study.

**Table 5:** Risk factors associated with western Thailand *Plasmodium falciparum* positivity (multiple failure time model).

Risk factor	aHR	*P*
Kanchanaburi	1.69 (0.54–5.26)	0.36
Season (May–September)	2.12 (1.03–4.36)	0.04

Age group (years; ref: 0–6)		
7–12	4.15 (0.38–45.23)	0.12
13–17	4.54 (0.45–46.25)	
≥18	10 (1.1–91.05)	

Male	2.9 (0.73–11.52)	0.13
Work in agriculture^a^	0.36 (0.05–2.63)	0.32
Travel frequency to Myanmar^b^	1.08 (0.96–1.22)	0.18
Average bednet usage^b^, ^c^	1.04 (0.83–1.3)	0.76
IRS^a^	2.12 (0.77–5.82)	0.15
Previous clinical malaria^a^	0.96 (0.28–3.24)	0.95

aHR, adjusted hazard ratio; IRS, indoor residual spray.

a Status at enrolment.

b As a time-changing covariate (floating average at time of outcome).

c Average bednet usage was defined as the proportion of times a person had answered ‘yes’ to the question: ‘Did you sleep under a bednet last night?’.

## 4 Discussion

This study has confirmed the high degree of heterogeneity of malaria risk in Thai populations living along the Thailand – Myanmar boarder and identified important risk factors for *P. vivax* infection and disease. One key epidemiological parameter determined in this study is the _mol_FOB which reflects the rate of acquiring a new blood-stage clone of the parasite. Two genetic markers (PvMS2 and Pvmsp1F3) were used to genotype *P. vivax*. Because the combined *H*_e_ of the two loci was 0.89, approximately 10% of all new clones are expected to have been missed. This, together with the limited sensitivity of molecular genotyping (69.5% of samples were PvMS2-positive; 78.9% of samples were Pvmsp1F3-positive), would lead to an underestimation of the true _mol_FOB. These limitations are shared by our previous studies using the same methodology (Mueller et al., 2012, Koepfli et al., 2013, Hofmann et al., 2017). The analyses of risk factors, however, depends only on the relative values of the _mol_FOB.

Overall the prevalences of *P. falciparum* (0–1.3%) and *P. vivax* (1.7–4.2%) were similar to the 4.9–5.7% *Plasmodium* prevalence previously reported in Kanchanaburi and Ratchaburi in 2012 (Patel et al., 2014, Nguitragool et al., 2017). The majority of infections detected at each visit were asymptomatic and resolved naturally without developing into clinical malaria. Using the same dataset, we recently estimated the duration of blood-stage infection detectable by molecular genotyping to be 29 days for *P. vivax* and 135 days for *P. falciparum* (White et al., 2018).

The overall prevalence as well as the predominance of *P. vivax* infections reflect the current trend in Thailand and the Greater Mekong Subregion where *P. vivax* has become the predominant malaria species (Baum et al., 2015, Imwong et al., 2015, Nguitragool et al., 2017). The *P. vivax* pre-dominance is less pronounced among the clinical/febrile cases (Pv: 79%) than asymptomatic infections (Pv: 91.5%). The predominance was most striking in the number of genetically distinct blood-stage infections acquired during follow-up (_mol_FOB) with only 7.4% of all newly acquired infections due to *P. falciparum*, resulting in an average _mol_FOB of 0.02 and 0.24 new infections per person-year for *P. falciparum* and *P. vivax,* respectively. The proportion of *P. vivax* infection relative to *P. falciparum* was much higher in our cohort than among clinical cases recorded by the Thai health system (ThaiDDC, 2016, WHO, 2016), indicating that the true burden of (mostly asymptomatic) *P. vivax* infections in Thailand may be substantially higher than reported.

The risk of *P. vivax* infection was highly heterogeneous in our study sites, which are fairly typical of areas along the Thailand-Myanmar border where the landscape is mountainous, agriculture and forest harvesting are the means of living, and cross-border migrant workers are common. Only 15% of the 999 study participants tested positive during the study, and people who were infected at multiple visits accounted for >80% of all infections. The observed concentration of infections in a small sub-population suggests that appropriate interventions targeted at high-risk individuals may be effective in accelerating malaria elimination.

In order to target malaria interventions more efficiently, it is essential to better understand the geographical, demographic, and behavioral risk factors for *Plasmodium* spp. infections in these Thai border communities. For *P. vivax*, the risk factors for higher parasite prevalence and _mol_FOB were very similar and included rainy season, aged 7–60 years, male gender, past experience of clinical malaria, being employed in agriculture, and travel to Myanmar. As areas with perennial transmission, our study sites received deltamethrin IRS twice each year according to the policy of the Thai Ministry of Public Health. Our analysis found that this intervention was associated with reduced infection risk. Thus, even though malaria infection risk was generally related to occupational exposure (as exemplified by male adults working in agriculture or in the forest and people visiting areas of higher transmission in Myanmar), the high risk in children 7–17 years of age and the association of IRS with protection indicate ongoing local *P. vivax* transmission, including in or near people’s houses. While the high risk in male adults and those regularly crossing the Myanmar border has been well established (Nguitragool et al., 2017), the association between IRS and reduced *P. vivax* positivity was a surprise in light of the predominantly outdoor biting preference of the malaria vectors in western Thailand (Muenworn et al., 2009, Tananchai et al., 2012, Tisgratog et al., 2012, Sriwichai et al., 2017). It provides a strong argument for continued support for the national IRS program in country.

The risk of suffering a clinical *P. vivax* episode was highest in children <7 years of age and decreased strongly with age, indicating that residents with higher risks of *P. vivax* infections did acquire substantial clinical immunity. Although it is well established that immunity to *P. vivax* is acquired more rapidly than to *P. falciparum* (Longley et al., 2016), it cannot be ruled out that at least part of the immunity observed in older age groups was acquired during earlier periods when transmission intensity was substantially higher (Phimpraphi et al., 2008). Although they are less infectious to mosquitoes than symptomatic infections (Kiattibutr et al., 2017), asymptomatic *P. vivax* infections are much more prevalent and may pose a particular challenge to malaria elimination in Thailand. Overall, the observed risk patterns indicate that residual transmission of *P. vivax* persists in our study areas.

There was only a small number of *P. falciparum-*positive cases detected during the study period. Due to this, for *P. falciparum* the risk factor analysis was limited to qPCR positivity. Only frequency of travel to Myanmar was found to be a statistically significant determinant for risk of *P. falciparum* infection. Although not statistically significant due to the profound lack of power, the multivariate analysis does indicate that the risk of *P. falciparum* was highest in male (adjusted hazard ratio (aHR) = 2.9) adults (aHR = 10.0) with an excess risk also during the rainy seasons and in areas where IRS had been conducted. The higher risk in people who traveled frequently across the border is a common feature of the residual burden both in this (Nguitragool et al., 2017) and other border regions of Thailand (Kitvatanachai et al., 2003, Bhumiratana et al., 2013). As found in neighboring Tak province, where *P. falciparum* malaria was four times more likely in recent migrants compared with Thai patients and correlated with *Anopheline* vector capture rates (Sriwichai et al., 2017), this indicates that importation of *P. falciparum* to Ratchaburi and Kanchanaburi contributed significantly to prevalence.

To our knowledge, this study is the first from a hypoendemic area to determine the _mol_FOB. Previous studies of *Plasmodium*_mol_FOB were restricted to Papua New Guinea where transmission intensity was much higher with _mol_FOB in the range of 5–14 new infections per person-year (Mueller et al., 2012, Koepfli et al., 2013, Hofmann et al., 2017). These previous studies demonstrated that in such a setting, acquisition of new *P. falciparum* clones was a major factor of clinical malaria in children (Mueller et al., 2012) and that high _mol_FOB likely contributed to rapid acquisition of immunity against *P. vivax* malaria (Koepfli et al., 2013). The _mol_FOB for both *P. falciparum* and *P. vivax* were at least 50-fold lower in the current study. This study thus represents a scenario at the opposite end of the malaria transmission spectrum.

Compared with parasite positivity, the _mol_FOB is a more direct measure of *P. falciparum* transmission. For *P. vivax*, the _mol_FOB is a combined measure of blood-stage infections arising from both new mosquito bites and relapses. Without the knowledge about the relative contributions of mosquito bites and relapses, it is difficult to estimate how much the _mol_FOB reflects transmission intensity. In this study, we found that the seasonal variation of parasite positivity closely followed that of the _mol_FOB (Fig. 2) for both parasite species. Risk factor analyses of the _mol_FOB and positivity of *P. vivax* also yielded similar results; all significant risk factors apparent in the _mol_FOB analysis were apparent in the analysis that used positivity. The close association between *P. vivax* prevalence and the _mol_FOB is consistent with the short duration (29 days) estimated for blood-stage infection in Thailand (White et al., 2018).

In summary, our study highlights the different challenges posed by *P. falciparum* and *P. vivax* to Thailand’s declared goal of eliminating local malaria transmission by 2025. In line with our earlier studies in Tak Province (Baum et al., 2015, Baum et al., 2016, Parker et al., 2015, Sriwichai et al., 2017), we have confirmed the continued presence of local *P. vivax* transmission in Thai villages along the western border. Most of the *P. vivax* infections were asymptomatic and often of very low density and would not have been detected by the standard surveillance methods based on microscopy and centered around passive case detection. Efficient elimination of *P. vivax* from Thailand will thus require additional novel strategies targeting this asymptomatic reservoir, including the silent hypnozoite reservoir (Robinson et al., 2015). On the other hand, *P. falciparum* infections were rare. With a large proportion of infection linked to recent travel to Myanmar, it will be important to strengthen malaria control measures on both sides of the border and to raise awareness of the travel-associated risk.
