# Supplementary material for: Highly heterogeneous residual malaria risk in western Thailand
Source: Int J Parasitol. 2019 May;49(6):455–62. doi: 10.1016/j.ijpara.2019.01.004 (PMC6996282; doi:10.1016/j.ijpara.2019.01.004)
Supplement: Supplementary data 1 [file mmc1.docx]

**Supplementary Tables**

This supporting information document contains univariate model estimates and back-selected models in which the statistically significant (based on a significance level of 0.05) variables were retained.

Supplementary Table S1. Univariate model estimates of factors associated with *Plasmodium vivax* _mol_FOB (negative binomial model) 2

Supplementary Table S2. Back-selected model of factors associated with *Plasmodium vivax* _mol_FOB (negative binomial model, based on a significance level of 0.05) 3

Supplementary Table S3. Univariate model estimates of factors associated with *Plasmodium vivax* positivity (multiple failure Cox proportional hazards model) 4

Supplementary Table S4. Back-selected model of factors associated with *Plasmodium vivax* positivity (multiple failure Cox proportional hazards model) 5

Supplementary Table S5. Univariate model estimates of factors associated with *Plasmodium vivax* clinical disease (multiple failure Cox proportional hazards model) 6

Supplementary Table S6. Back-selected model of factors associated with *Plasmodium vivax* clinical disease (multiple failure Cox proportional hazards model). This model is presented once with _mol_FOB and once without _mol_FOB as a covariate. 7

Supplementary Table S7. Univariate model estimates of factors associated with *Plasmodium falciparum* positivity (multiple failure Cox proportional hazards model). The most parsimonious model in this case is the univariate model for travel frequency to Myanmar. 8

## Supplementary Table S1. Univariate model estimates of factors associated with *Plasmodium vivax* _mol_FOB (negative binomial model)

| **Risk factor** | **IRR** | **95% CI** | ***P*>z** |
| --- | --- | --- | --- |
|  |  |  |  |
| Kanchanaburi | 2.71 | 1.17-6.29 | 0.020 |
|  |  |  |  |
| Age group (years; ref: 0-6) |  |  |  |
| 7-12 | 21.99 | 2.85-169.97 | <0.001 |
| 13-17 | 22.79 | 2.45-211.62 |  |
| 18-60 | 55.40 | 7.67-200.09 |  |
| >60 | 14.06 | 1.56-125.94 |  |
|  |  |  |  |
| Male | 2.197 | 1.24-3.90 | 0.007 |
| Frequency of travel to Myanmar (per visit/year) | 1.37 | 1.24-1.51 | <0.001 |
| Previous clinical malaria^a^ | 3.78 | 2.07-6.90 | <0.001 |
| House treated with IRS^a^ | 0.89 | 0.50-1.60 | 0.70 |
| Reported bednet possession^a^ | 1.08 | 0.43-2.67 | 0.87 |
| Work in agriculture^a^ | 4.32 | 2.53-7.66 | <0.001 |

^a^At first visit (May 2013).

IRR, incidence rate ratio; CI, confidence interval; IRS, indoor residual spray.

## Supplementary Table S2. Back-selected model of factors associated with *Plasmodium vivax* _mol_FOB (negative binomial model, based on a significance level of 0.05)

| **Risk factor** | **IRR** | **95% CI** | ***P*>z** |
| --- | --- | --- | --- |
|  |  |  |  |
| Age group (years; ref: 0-6) |  |  |  |
| 7-12 | 21.75374 | 3.28-144.48 | <0.001 |
| 13-17 | 19.21961 | 2.3-160.64 |  |
| 18-60 | 15.89609 | 2.2-115.04 |  |
| >60 | 4.267298 | 0.48-37.86 |  |
|  |  |  |  |
| Male | 2.00 | 1.18-3.38 | 0.01 |
| Frequency of travel to Myanmar (per visit per year) | 1.17 | 1.07-1.28 | <0.001 |
| Previous clinical malaria^a^ | 2.17 | 1.11-4.27 | 0.024 |
| Work in agriculture^a^ | 2.28 | 1.06-4.9 | 0.034 |

^a^At first visit (May 2013).

IRR, incidence rate ratio; CI, confidence interval.

## Supplementary Table S3. Univariate model estimates of factors associated with *Plasmodium vivax* positivity (multiple failure Cox proportional hazards model)

| **Risk factor** | **HR** | ***P*** |
| --- | --- | --- |
|  |  |  |
| Kanchanaburi | 2.33 (1.27-3.56) | 0.013 |
| Season (May-September) | 3.07 (1.96-4.80) | <0.001 |
|  |  |  |
| Age group (years; ref: 0-6) |  |  |
| 7-12 | 3.83 (0.99-14.71) | <0.001 |
| 13-17 | 7.15 (1.85-27.6) |  |
| 18-60 | 9.00 (2.72-29.79) |  |
| >60 | 2.57 (0.59-11.15) |  |
|  |  |  |
| Male | 2.97 (1.77-4.98) | <0.001 |
| Work in agriculture^a^ | 3.33 (2.07- 5.34) | <0.001 |
| Frequency of travel to Myanmar^b^ | 1.15 (1.10-1.20) | <0.001 |
|  |  |  |
| Average bednet usage^b,c^ | 0.95 (0.91-1.00) | 0.05 |
| House treated with IRS^a^ | 0.79 (0.48-1.32) | 0.37 |
| Previous clinical malaria^a^ | 3.05 (1.83-5.096) | <0.001 |

^a^Status at enrolment; ^b^as time-changing covariate (average observed at time of outcome); ^c^average bednet usage was defined as the proportion of times a person had answered `yes’ to the question: `Did you sleep under a bednet last night’ during active case detection.

HR, hazard ratio; IRS, indoor residual spray.

## Supplementary Table S4. Back-selected model of factors associated with *Plasmodium vivax* positivity (multiple failure Cox proportional hazards model)

| Risk factor | **HR** | ***P*** |
| --- | --- | --- |
| Kanchanaburi | 1.57 (1.23-2.53) | 0.031 |
| Season (May-September) | 3.42 (2.2-5.31) | <0.001 |
|  |  |  |
| Age group (years; ref: 0-6) |  |  |
| 7-12 | 3.64 (0.95-13.97) | <0.001 |
| 13-17 | 5.01 (1.24-20.23) |  |
| 18-60 | 3.69 (1.03-13.23) |  |
| >60 | 0.99 (0.22-4.49) |  |
|  |  |  |
| Male | 2.32 (1.37-3.94) | 0.002 |
| Work in agriculture^a^ | 2.01 (1.14-3.56) | 0.016 |
| Frequency of travel to Myanmar^b^ | 1.06 (1.01-1.11) | 0.010 |
| House treated with IRS^a^ | 0.47 (0.28-0.79) | 0.005 |
| Previous clinical malaria^a^ | 1.87 (1.14-3.08) | 0.014 |

^a^Status at enrolment; ^b^as time-changing covariate (average observed at time of outcome).

HR, hazard ratio; IRS, indoor residual spray,.

## Supplementary Table S5. Univariate model estimates of factors associated with *Plasmodium vivax* clinical disease (multiple failure Cox proportional hazards model)

| **Risk factor** | **Adjusted for _mol_FOB^a^** | |  | **Univariate** | |
| --- | --- | --- | --- | --- | --- |
|  | **aHR** | ***P*** |  | **HR** | ***P*** |
| *P. vivax* _mol_FOB^b^ |  |  |  | 1.53 (1.44-1.64) | <0.001 |
|  |  |  |  |  |  |
| Kanchanaburi | 1.45 (0.56-3.690) | 0.45 |  | 0.73 (0.28-1.93) | 0.53 |
| Season (May-September) | 12.23 (1.89-79.36) |  |  | 22.76 (1.48-350.4) | 0.03 |
|  |  |  |  |  |  |
| Age group (years; ref: 0-12)^c^ |  |  |  |  |  |
| 13-17 | 1.29 (0.31-5.34) | 0.41 |  | 1.9 (0.57-6.36) | 0.53 |
| 18-60 | 0.5 (0.14-1.76) |  |  | 1.33 (0.49-3.61) |  |
| >60 | 1.5 (0.46-4.96) |  |  | 1.67 (0.42-6.68) |  |
|  |  |  |  |  |  |
| Male | 2.55 (1.13-5.77) | 0.025 |  | 2.58 (1.09-6.12) | 0.031 |
| Work in agriculture^d^ | 1.21 (0.42-3.46) | 0.75 |  | 2.97 (1.34-6.58) | <0.008 |
| Travel frequency to Myanmar^b^ | 1.02(0.91-1.14) | 0.73 |  | 1.10 (1.01-1.19) | 0.024 |
|  |  |  |  |  |  |
| Average bednet usage ^b,e^ | 0.82 (0.76-0.88) | <0.001 |  | 0.87 (0.82-0.91) | <0.001 |
| IRS^4^ | 1.46 (0.58-3.67) | 0.42 |  | 1.7 (0.69-4.2) | 0.25 |
| Previous clinical malaria^d^ | 1.75 (0.68-4.52) | 0.25 |  | 2.95 (1.25-6.97) | 0.013 |

^a^_mol_FOB was the only other variable included into the model. ^b^as time-changing covariate (floating average at time of outcome); ^c^The age groups were further aggregated when compared with Table S3, as some age groups did not contain any positive outcomes; ^d^status at enrolment; ^e^average bednet usage was defined as the proportion of times a person had answered ‘yes’ to the question: ‘Did you sleep under a bednet last night’.

aHR, adjusted hazard ratio; HR, hazard ratio; IRS, indoor residual spray.

## Supplementary Table S6.Back-selected model of factors associated with *Plasmodium vivax* clinical disease (multiple failure Cox proportional hazards model). This model is presented once with _mol_FOB and once without _mol_FOB as a covariate.

| **Risk factor** | **Model with _mol_FOB** | |  | **Model w/o _mol_FOB** | |
| --- | --- | --- | --- | --- | --- |
|  | **aHR** | ***P*** |  | **HR** | ***P*** |
| *P. vivax* _mol_FOB^a^ | 1.58 (1.45-1.72) | <0.001 |  |  |  |
|  |  |  |  |  |  |
| Season (May-September) | 9.55 (1.32-68.92) | 0.025 |  | 18.74 (1.57-223.47) | 0.020 |
| Age group (years; ref: 0-12)^b^ |  |  |  |  |  |
| 13-17 | 0.71 (0.14-3.58) | 0.005 |  | 0.7 (0.18-2.8) | 0.008 |
| 18-60 | 0.21 (0.07-0.64) |  |  | 0.25 (0.11-0.58) |  |
| >60 | 0.45 (0.15-1.37) |  |  | 0.32 (0.09-1.14) |  |
|  |  |  |  |  |  |
| Male | 2.28 (1.06-4.89) | 0.035 |  |  |  |
| Work in agriculture^c^ | 3.59 (1.6-8.03) | 0.002 |  | 5.18 (2.52-10.64) | <0.001 |
|  |  |  |  |  |  |
| Average bednet usage ^a,d^ | 0.83 (0.77-0.91) | <0.001 |  | 0.89 (0.83-0.95) | <0.001 |
| Previous clinical malaria^c^ |  |  |  | 2.53 (1.23-5.19) | 0.012 |

^a^As time-changing covariate (floating average at time of outcome); ^b^The age groups were further aggregated when compared with Supplementary Table S3, as some age groups did not contain any positive outcomes; ^c^status at enrolment; ^d^average bednet usage was defined as the proportion of times a person had answered ‘yes’ to the question: ‘Did you sleep under a bednet last night’.

aHR, adjusted hazard ratio; HR, hazard ratio.

## Supplementary Table S7. Univariate model estimates of factors associated with *Plasmodium falciparum* positivity (multiple failure Cox proportional hazards model). The most parsimonious model in this case is the univariate model for travel frequency to Myanmar.

| **Risk factor** | **HR** | ***P*** |
| --- | --- | --- |
|  |  |  |
| Kanchanaburi | 2.43 (0.73-8.33) | 0.147 |
| Season (May-September) | 1.60 (0.69-3.71) | 0.271 |
|  |  |  |
| Age group (years; ref: 0-6) |  |  |
| 7-12 | 4.04 (0.36-44.97) | 0.31 |
| 13-17 | 4.47 (0.44-44.88) |  |
| ≥18 | 6.64 (0.83-53.24) |  |
|  |  |  |
| Male | 2.52 (0.65-9.85) | 0.182 |
| Work in agriculture^a^ | 1.06 (0.29-3.84) | 0.929 |
| Travel frequency to Myanmar^b^ | 1.11 (1.01-1.22) | 0.033 |
|  |  |  |
| Average bednet usage^b,c^ | 1.01 (0.82-1.25) | 0.941 |
| IRS^2^ | 2.37 (0.79-7.15) | 0.124 |
| Previous clinical malaria^a^ | 1.57 (0.48-5.11) | 0.455 |

^a^Status at enrolment; ^b^as time-changing covariate (floating average at time of outcome); ^c^average bednet usage was defined as the proportion of times a person had answered `yes’ to the question: ‘Did you sleep under a bednet last night.’

HR, hazard ratio; IRS, indoor residual spray.
